# Regional Variation on Rates of Bronchopulmonary Dysplasia and Associated Risk Factors

**DOI:** 10.5402/2012/685151

**Published:** 2012-07-05

**Authors:** María Ximena Rojas, Mario Augusto Rojas, Juan Manuel Lozano, Martín Alonso Rondón, Laura Patricia Charry

**Affiliations:** ^1^Department of Clinical Epidemiology and Biostatistics, School of Medicine, Pontificia Universidad Javeriana, Bogotá 110001, D.C., Colombia; ^2^Division of Neonatology, Wake Forest University School of Medicine, Winston-Salem, NC, USA; ^3^Division of Research and Information, College of Medicine, Florida International University, Miami, FL, USA

## Abstract

*Background*. An abnormally high incidence (44%) of bronchopulmonary dysplasia with variations in rates among cities was observed in Colombia among premature infants. *Objective*. To identify risk factors that could explain the observed high incidence and regional variations of bronchopulmonary dysplasia. Study Design. A case-control study was designed for testing the hypothesis that differences in the disease rates were not explained by differences in city-of-birth specific population characteristics or by differences in respiratory management practices in the first 7 days of life, among cities. *Results*. Multivariate analysis showed that premature rupture of membranes, exposure to mechanical ventilation after received nasal CPAP, no surfactant exposure, use of rescue surfactant (instead of early surfactant), PDA, sepsis and the median daily FIO_2_, were associated with a higher risk of dysplasia. Significant differences between cases and controls were found among cities. Models exploring for associations between city of birth and dysplasia showed that being born in the highest altitude city (Bogotá) was associated with a higher risk of dysplasia (OR 1.82 95% CI 1.31–2.53). *Conclusions*. Bronchopulmonary dysplasia was manly explained by traditional risk factors. Findings suggest that altitude may play an important role in the development of this disease. Prenatal steroids did not appear to be protective at high altitude.

## 1. Introduction

Despite all the advances in the care of premature infants with respiratory distress syndrome (RDS), including the use of antenatal steroids and early management with surfactant, bronchopulmonary dysplasia (BPD) continues to be a major cause of chronic morbidity among this population. There are large variations in the incidence and severity of this disease. According to the National Institutes of Health of USA (NICHD) consensus [1], mild BPD is defined as a need for supplemental oxygen for ≥28 days at 36 weeks postmenstrual age (wPMA) or discharge, moderate BPD as supplemental oxygen for ≥28 days plus treatment with <30% oxygen at 36 wPMA, and severe BPD as supplemental oxygen for ≥28 days plus ≥30% oxygen and/or positive pressure at 36 wPMA. Currently, the estimated incidence of BPD defined as need for supplemental oxygen at 36 wPMA in the United States is approximately 30% for premature infants with a birth weight <1000 grams and <7% in infants with a birth weight >1250 grams or who were at least 30 weeks of gestation at birth [1, 2]. 

Previous epidemiological studies have identified prematurity, oxygen toxicity, and mechanical ventilation as major risk factors associated with BPD [3–7]. Additional risk factors include perinatal or postnatal infection/inflammation [8–12], pulmonary edema resulting from patent ductus arteriosus (PDA) [12, 13] or excess fluid administration [7, 14, 15], vitamin A deficiency [16], early adrenal insufficiency [17], and, more recently, genetic polymorphism [18].

There is little information about trends in the epidemiology and pathogenesis of BPD in developing countries. The most recent report of the incidence of BPD in Latin America comes from the NEOCOSUR Neonatal Group study in 1,825 very-low-birth-weight (VLBW) infants born in sixteen hospitals from Argentina, Chile, Paraguay, Peru, and Uruguay [19]. The authors found an incidence of BPD (oxygen requirement at 28 days of life with chronic radiographic changes) of 24.4%. A randomized controlled trial of early bubble nasal continuous positive airway pressure (nCPAP) and surfactant in premature infants conducted in three different cities in Colombia [20] found an incidence of BPD (defined as supplemental oxygen requirement at 36 wPMA) twice as large (44%) as the one observed in less mature premature infants in developed countries or in the NEOCOSUR study. The Colombian trial also revealed significant variations in BPD rates among participating cities: Bogotá 50%, Cali 18%, and Bucaramanga 13%. These cities have different characteristics, the most important being altitude: Bogotá is 2600 meters above sea level (masl), Bucaramanga 959 masl, and Cali 956 masl.

We conducted this study with the aim of identifying environmental, maternal, infant, and therapeutic risk factors associated with BPD in Colombia. We also tested the hypothesis that differences in BPD rates were not explained by differences in city-of-birth-specific population characteristics or by differences in respiratory management practices during the first seven days of infants' life among cities.

## 2. Materials/Subjects and Methods

This is a nested case-control study based on data collected as part of a multicenter randomized controlled trial carried out by the Colombian Neonatal Research Network in eight neonatal intensive care units (NICUs) located in three cities (Bogotá, Bucaramanga, and Cali) in Colombia. A detailed description of this trial has been published [20]. Briefly, premature infants born between 27 and 31 weeks of gestation with clinical evidence of respiratory distress during the first hour of life, and who did not require intubation as part of their initial resuscitation and stabilization, were placed on bubble nCPAP and then randomized to receive very early surfactant therapy through transient intubation followed by nCPAP or to expectant management on nCPAP alone. A total of 279 premature infants were enrolled in the trial from January 1, 2004 to December 31, 2006. All study sites provided comprehensive continuous care for critically ill neonates and were staffed by trained nurses and specialized physicians with fully equipped and modern neonatal units. Prenatal and neonatal data were collected prospectively until death or discharge. Additional data were collected at 36 wPMA.

The neonatal survival for the population included in the RCT was 90.7%. Neonatal mortality rates were similar among cities. For the present study, all analyses were limited to infants who survived to 36 wPMA. Bronchopulmonary dysplasia was defined as the need for supplemental oxygen for ≥28 days at 36 wPMA [1]. The target oxygen arterial saturation (SaO_2_) was 92% for infants treated in Bogota and 96% for those treated in other cities. Given the high altitude in Bogotá it was expected that SaO_2_ would be lower than in infants at lower altitudes [21, 22]. Neonates who fell under this definition were considered as cases. Since we used an epidemiological definition for BPD and we did not have the radiographic findings to confirm BPD cases, to avoid misclassification bias “controls” were all infants who were not receiving supplemental oxygen at 36 wPMA and had required <20 days of oxygen supplementation or had not required oxygen supplementation at all. Cases and controls were compared to identify risk factors associated with BPD. Relevant exposure data included maternal and infant perinatal characteristics, infant postnatal diagnosis, and respiratory management practices. *Preterm premature rupture of membranes* (PPROM) was defined as >12 hours, *use of antenatal steroids* as the administration of a complete course (two 12 mg intramuscular doses of betamethasone within 24 hours) of maternal steroids at least 24 hours before delivery, *confirmed chorioamnionitis* as a positive amniotic fluid culture, and *suspected chorioamnionitis* as maternal fever during labor and fetal tachycardia. *Gestational age* (GA) at birth was estimated using the last menstrual period date; when GA was inconsistent with the physical examination, the Ballard score was used [23]. *Small for gestational age *(SGA) was defined as a birth weight <10th percentile for age [24]. *PDA *was confirmed by echocardiography and subsequently treated with indomethacin or surgical ligation. *Sepsis* was defined as a clinical deterioration with temperature instability, recurrent apnea, and at least one of the following indications of altered organ function: lethargy, hypoxemia, and increased serum lactate level [25]. Grades* III and IV IVH* were identified by head ultrasound using the Papile et al. criteria [26]. *Air leak syndrome* included radiological evidence of pneumothorax, pneumomediastinum, or pulmonary interstitial emphysema. The* Score for Neonatal Acute Physiology (SNAP II)* on the day of admission was calculated from clinical data collected prospectively during the first day of life [27]. Respiratory management variables included *type of ventilatory support* received: (a) *only nCPAP* when infants were placed on nCPAP without subsequent mechanical ventilation (MV) during their NICU stay, (b) *nCPAP + MV *when infants on nCPAP met treatment failure criteria and required MV. *Length of MV* was categorized in ≥7 days or <7 days. *Exposure to surfactant* was divided into three categories: (a)  *no surfactant exposure* included all infants who did not receive surfactant during the trial, (b)  *very early surfactant* included infants administered surfactant within the first hour of life, and (c)  *rescue surfactant* included infants administered surfactant after the first hour of life. Exposure to oxygen supplementation was measured by *fraction of inspired oxygen* (FIO_2_) registered daily.

Demographic and clinical characteristics of the study population were summarized. To identify differences in the distribution of studied variables between cases and controls, the statistical analysis included hypothesis testing using the Pearson Chi-square test for categorical variables and the Students *t*-test for continuous variables. To explore associations between studied variables and BPD, crude and adjusted odds ratios (ORs) with 95% confidence intervals (95% CIs) were estimated in a bivariate analysis, followed by a multivariate regression analysis using a log-binomial generalized linear model that included all significant variables in a manual forward stepwise approach. The Wilcoxon rank sum test was used to compare the median values between groups and the Generalized Cochrane Mantel-Haenszel test to assess the statistical significance of associations between categorical variables.

To determine differences in population characteristics or in respiratory management variables during the first week of life that could explain the differences in BPD rates among cities, a descriptive analysis of selected variables was performed by *city of birth*, followed by a bivariate analysis to identify variables mainly associated with BPD in each city. To identify possible differences in the distribution of studied variables between three cities among cases of BDP, the statistical analysis included hypothesis testing using the Pearson Chi-square test for categorical variables and the Analysis of Variance or Kruskall Wallis test for continuous variables. Multivariate analyses were then conducted to explore associations between *city of birth* and BPD while controlling for differences in population demographic characteristics, postnatal diagnosis, and respiratory management variables during the first seven days of life. With the final sample of cases and controls, the power calculation for the research hypothesis tested was 82%, according to the following parameters: incidence of BPD 30%, type I error probability of 0.05, and expected size effect (odds ratio, OR) of 2.0. Results of all multivariate analyses are expressed as odds ratios (ORs) with their corresponding 95% CI. All analyses were carried out using the SAS program (SAS Institute, Cary, NC).

## 3. Results

A total of 216 (77.42%) infants survived to 36 wPMA, and four were excluded from the analysis due to missing data or because they did not meet the case or control definitions. The final analysis included 212 infants; 64 (30%) met the definition of BPD.

### 3.1. Identification of Risk Factors for BPD in the Whole Population

Mean GA and birth weight were lower in the BPD group (Table 1); no differences were observed in other perinatal characteristics between cases and controls. Tables 2 and 3 present the results of bivariate analysis controlling for GA at birth. *Use of rescue surfactant, no surfactant exposure, diagnosis of PDA, chorioamnionitis, and confirmed or suspected PPROM *were variables independently associated to BPD.

Following a stepwise forward approach, the initial logistics model showed that *PPROM*, *nCPAP + MV, no surfactant exposure, *rescue surfactant, PDA, median daily FIO_2_ (sub index is not allowed in this website), *and sepsis* were associated with a higher risk of BPD. When *city of birth* was introduced into the model as a control variable, we observed that *median daily *FIO_2_ and *PDA* lost statistical significance, suggesting the presence of an interaction. We conducted a series of multivariate models testing for interactions between the variables: *city of birth, median daily* FIO_2_ *;* *sepsis and PDA*. The only significant interaction observed was between the variables “*Bogotá as* *city of birth” *and* “median daily *FIO_2_ *”* The results of the final logistic regression model controlling this interaction are showed in Table 4. “*Bogotá as* *city of birth” *was the most significant independent variable associated with BPD; other variables associated with BPD in the initial model remained significantly associated (Table 4).

### 3.2. BPD Rates and City-Specific Differences

The distribution of maternal and infant perinatal characteristics, infant postnatal diagnosis, and respiratory management practices in the first seven days of the infants' life was similar in all cities (Table 5); however, there are statistically significant differences between cases and controls among cities.

The proportion of BPD cases in infants whose mothers *received antenatal steroids* was significantly higher in Bogotá than in any other city. In Bucaramanga, infants with BPD had lower birth weights than infants in Cali or Bogotá. Infants born in Bogotá that required MV after nCPAP *(nCPAP+MV)* and those treated with *rescue surfactant* or *no surfactant exposure* had a higher incidence of BPD compared to infants of similar characteristics born in Cali or Bucaramanga. Infants born in Bogotá had higher median values of daily FIO_2_ compared to Bucaramanga and Cali, and infants diagnosed with BPD in Bogotá received higher concentrations of daily supplemental oxygen than controls, while BPD cases in Cali and Bucaramanga did not.

In relation to postnatal diagnosis during the first 7 days of life, the diagnosis of PDA was higher in Bucaramanga, but the largest proportion of BPD cases in infants with PDA was observed in Bogotá. The diagnosis of sepsis was also more frequent in Bucaramanga and Cali, but the largest proportion of BPD cases among infected infants was seen in Bogotá, followed by Cali and Bucaramanga (*P* < 0.0001).

Bivariate analysis stratified by *city of birth* (Table 5) showed the following: for infants born in Bogotá, the diagnosis of* sepsis* in the first seven days of life and the incremental levels of* daily median *FIO_2_ as variables mainly associated with a higher risk of BPD; for infants born in Cali, a*ir leak syndrome, PPROM, *and* PDA *in the first seven days of life were the variables associated with BPD, while in Bucaramanga *PPROM *was the only significant risk factor associated with BPD.

To explore for associations between *city of birth* and BPD, we generated a logistics model controlling potential confounders. Multivariate results are shown in Table 6; *Bogotá as city of birth* was strongly associated with an increased risk of developing BPD (OR 1.82 95%CI 1.31–2.53). Other variables in the model associated with a higher risk of BPD were *rescue surfactant*, *no surfactant exposure,nCPAP* + *MV*, *median daily *FIO_2_, *PDA,* and *sepsis*.

## 4. Discussion

In this study population, the incidence of BPD was 30%. This rate is higher than the average rates reported in populations with similar gestational ages in developed countries [1, 2, 5–7]. Our results showed that infants born in Bogotá had nearly twice the risk of developing BPD than infants born in Bucaramanga or Cali, independently of differences in maternal, infant, and therapeutic risk factors. Additionally, Bogotá showed the highest rates of BPD cases associated with the presence of air leak syndrome, exposure to MV, rescue surfactant or no surfactant exposure, PDA, and sepsis, when compared to other cities. It also had the highest values of daily supplemental oxygen to reach the target SaO_2_ among all the cities. Exposure to antenatal steroids did not appear to protect infants born in Bogotá from developing BPD.

Because Bogotá is located a higher altitude (more than 2600 masl) than Bucaramanga and Cali, these results could suggest that altitude may play an important role in the pathogenesis of BPD in Colombia. There are few publications on altitude-related disease and pulmonary hemodynamics in pediatric populations, and the altitude where an infant is born has not clearly been proven to be associated with the development of BPD. The effect of living at high altitudes (>2500 masl) on lung diffusion capacity and pulmonary hemodynamics has been described in highland children [28]. As a result of the low partial pressure of oxygen in the environment, oxygen uptake into the lungs is enhanced by increases in minute ventilation, lung compliance, and pulmonary diffusion. The decreased partial pressure of oxygen in the lungs of highland children has also been associated with higher pulmonary artery pressures [28–31]. Several investigators have also found that functional closure of the ductus arteriosus in the newborn is delayed at high altitudes as a consequence of increased pulmonary vascular pressures [32–35]. The transition from oxygenation via the placenta to oxygenation across the formerly fluid-filled lungs is especially precarious in a low-oxygen environment [36]; with the onset of ventilation immediately after birth, the oxygen tension in the alveolus and pulmonary capillaries of neonates may not increase as expected, resulting in postnatal persistence of fetal circulation [36]. In the absence of pulmonary disorders, normal neonates may experience frequent episodes of arterial oxygen desaturations and hypoxia during the first week of life. Studies comparing healthy infants born at high altitudes (>3100 masl) with infants born at sea level have shown that a week after birth the SaO_2_ of high-altitude infants declines, whereas SaO_2_ gradually rises after birth or remains constant over time in infants born at sea level [32]. As a result of these events, it is possible that premature infants born at high altitudes have a prolonged transition period and early dependency on high concentrations of oxygen and ventilatory support compared to their counterparts born at lower altitudes. The presence of RDS would enhance ventilation perfusion mismatch leading to more hypoxia, oxygen dependency, oxidative stress, and higher levels of ventilatory support, increasing the risk and severity of BPD. It is also possible that the observed dependency on supplemental oxygen in our population of premature infants is the result of physiological oxygen dependency and not BPD. This may be due to the fact that the definition used for BPD does not take into account clinical or radiographic changes.

The association between PDA and BPD has previously been documented [4, 12, 37]. It is also possible that the prolonged closure of the PDA may play a role in the pathogenesis of BPD, especially as the pulmonary vascular resistance begins to drop, but we cannot answer this question because we did not assess the duration of the PDA, the presence of pulmonary hypertension, or the presence of pulmonary edema or hemorrhage in this population of infants. Likewise, we did not assess the effect of fluid intake on the development of PDA and BPD because this information was not collected in the initial trial [7, 14, 37, 38]. Previous studies have demonstrated the relationship between the presence of late-onset sepsis, PDA, and the development of BPD [12, 37]. Our study suggests that altitude enhances their negative effects and other traditional risk factors associated with BPD [38]. In our population, *PPROM* was found to be a significant risk factor for BPD while chorioamnionitis was not. This finding could be explained in part because amniocentesis and amniotic culture fluids were not taken routinely in all participating centers as part of the workup for suspected chorioamnionitis in mothers with preterm labor or premature rupture of membranes. The association of BPD with preterm labor and PPROM has been well documented in the medical literature [39].

Finally, our study emphasizes the need to minimize MV exposure and offer surfactant replacement therapy within the first hour of life to infants with RDS on NCPAP in order to decrease the incidence of BPD in this population [20, 40, 41].

To summarize, this study suggests that altitude may be an important risk factor associated with increased supplemental oxygen dependency and the development of BPD. Future studies need to determine whether altitude plays a role in the pathophysiology of BPD or if it is just a marker for physiologic oxygen demands. They also need to use a more accurate definition for BPD, measuring not only the need of supplemental oxygen but also the radiographic findings and clinical signs.

## Figures and Tables

**Table 1:** Demographic characteristics of population studied by cases and controls.

	All infants	BPD		
		Cases	Controls	*P*
	*n* = 212	*n* = 64	*n* = 148	
Maternal age, y, mean ± (SD)	27.2 (6.5)	26.1 (6.0)	27.3 (6.6)	0.205
Male (%)	108 (50.9)	34 (53.1)	74 (50.0)	0.676
Gestational age at birth, wPMA, mean ± (SD)	29.2 (1.3)	28.6 (1.2)	29.3 (1.3)	0.001
Birth weight, grams, mean ± (SD)	1271.2 (283.3)	1155.3 (263.0)	1306.5 (284.2)	0.000
SGA, *n* (%)	12 (5.7)	3 (4.7)	9 (6.1)	0.688
APGAR score 5 min, median (IR)	9 (8-9)	9 (8-9)	9 (8-9)	0.228
SNAP-II, score, mean ± (SD)	5.7 (9)	7.6 (10.5)	5.4 (8.6)	0.102

BPD: bronchopulmonary dysplasia, wPMA: weeks postmenstrual age, SGA: small for gestational age, SNAP-II: score for neonatal acute physiology, SD: standard deviation, IR: Interquartile range (difference between the value at 75% of cases, and the value at 25% of cases).

**Table 2:** Distribution of maternal characteristics by BPD status.

Characteristics	All infants	BPD					
		Cases	Controls	*P*	OR^∗^		95% CI
	*n* = 212	*n* = 64	*n* = 148				
Maternal characteristics							
Mean age, *n* (%)							
<18 years	15 (7.1)	5 (7.8)	10 (6.8)		1.08	0.38	3.08
18–35 years	173 (81.6)	52 (81.3)	121 (81.8)	0.959	1.00		
>35 years	24 (11.3)	7 (10.9)	17 (11.5)		0.97	0.50	1.88
Type of delivery, *n* (%)							
Vaginal	35 (16.5)	12 (18.8)	23 (15.5)	0.563	1.00		
Cesarean	177 (83.5)	52 (81.3)	125 (84.5)		0.86	0.51	1.43
Multiple gestation *n* (%)	22 (10.4)	6 (9.4)	16 (10.8)	0.753	0.89	0.44	1.83
Antenatal steroids, *n* (%)	184 (86.8)	56 (87.5)	128 (86.5)	0.072	1.32	0.63	2.75
PPROM, *n* (%)	76 (35.8)	30 (46.9)	46 (31.2)	0.028	1.58	1.06	2.36
Duration of rupture of membranes, *n* (%)							
<24 h	35 (46.1)	12 (40.0)	23 (50.0)	0.275	1.00		
24–48 h	14 (18.4)	5 (16.7)	9 (19.6)		0.76	0.14	4.02
48–72 h	8 (10.5)	2 (6.7)	6 (13.0)		1.76	0.52	5.94
>72 h	19 (25.0)	11 (36.7)	8 (17.4)		1.04	0.45	2.41
Chorio confirmed, *n* (%)	11 (5.2)	4 (6.5)	7 (4.7)	0.647	1.22	0.54	2.74
Chorio confirmed or suspected, *n* (%)	39 (18.4)	17 (26.6)	22 (14.9)	0.044	1.60	1.04	2.47

Chorio: chorioamnionitis, PPROM: preterm premature rupture of membranes.

**Table 3:** Distribution of infant characteristics by BPD status.

Characteristics	All infants	BPD					
		Cases *n* = 64	Controls *n* = 48	*P*	OR^∗^	95% CI	
Sex, *n* (%)							
Male	108 (50.9)	34 (53.1)	74 (50.0)	0.676	1.10	0.73	1.66
Female	104 (49.1)	30 (46.9)	74 (50.0)		1.00		
Gestational age, *n* (%)							
27–29 weeks	114 (53.8)	40 (62.5)	74 (50.0)	0.094	1.43	0.93	2.20
30-31 weeks	98 (46.2)	24 (37.5)	74 (50.0)		1.00		
Birth weight, *n* (%)							
<1000 g	37 (17.4)	14 (21.9)	23 (15.5)	0.301	1.70	0.86	3.38
1000–1500 g	130 (61.3)	40 (62.5)	90 (60.8)		1.30	0.76	2.53
>1500 g	45 (21.2)	10 (15.6)	35 (23.6)		1.00		
SGA *n* (%)	12 (5.7)	3 (4.7)	9 (6.1)	0.687	0.82	0.30	2.23
Apgar score <5 at 5 min, *n* (%)	2 (0.94)	0 (0.0)	2 (1.35)	0.350	1.00	0.82	1.21
Mean SNAP II, *n* (%)							
0 to 10	143 (67.4)	102 (68.9)	41 (64.0)				
11 to 19	54 (5.5)	36 (24.3)	18 (28.1)	0.381			
>20	15 (7.1)	10 (6.8)	5 (7.2)				
PDA (%)	64 (30.2)	30 (46.9)	34 (23.0)	0.001	2.04	1.38	3.03
Air Leak Syndrome (%)	12 (5.7)	6 (9.4)	6 (4.0)	0.124	1.72	0.94	3.16
Sepsis (%)	109 (51.4)	39 (60.9)	70 (47.3)	0.068	1.47	0.98	2.38
IVH grade III or IV (%)	4 (1.90)	1 (1.6)	3 (2.0)	0.820	0.83	0.15	4.56
Ventilatory Support (%)							
nCPAP only	162 (76.4)	37 (57.8)	125 (84.5)	0.000	1		
nCPAP + MV	50 (23.6)	27 (42.2)	23 (15.5)		2.36	1.61	3.46
LOMV ≥ 7 days (%)^∗^	15 (30.0)^∗^	8 (29.6)	7 (30.4)		1.12	0.68	1.84
Use of Surfactant (%)							
Very early	106 (50.0)	20 (31.2)	86 (58.1)	0.000	1		
Rescue	27 (12.7)	18 (28.1)	9 (6.1)		6.16	2.53	15.02
No surfactant	79 (37.3)	26 (40.6)	53 (35.8)		3.53	2.19	5.69
FIO_2_ (median values)	0.53	0.52	0.52	0.102			

Deaths before 36 wPMA are excluded. BPD: bronchopulmonary dysplasia, SGA: small for gestational age, SNAP-II: score for neonatal acute physiology, nCPAP: nasal continuous positive airway pressure, MV: mechanical ventilation, LOMV: length of mechanical ventilation, PDA: patent ductus arteriosus.

**Table 4:** Multivariate analysis predicting BPD for the entire cohort controlled by city of birth *N* = 212.

Variables	OR	95% CI	
Preterm premature rupture of membranes	1.17	1.05	1.31
Ventilatory support (nCPAP + MV)	1.18	1.01	1.38
Use of surfactant (rescue)	1.63	1.28	2.08
Use of surfactant (no surfactant)	1.37	1.12	1.67
City of birth (Bogotá)^1^	1.9	1.46	2.49
FIO_2_^1^ (median value)	1.58	1.11	2.26
PDA	1.14	1.01	1.28
Sepsis	1.12	1.01	1.26

^1^OR adjusted by interaction term between Bogota and FIO_2_.

**Table 5:** Variables assessed by city of birth for all infants studied and for BPD cases.

Characteristics	Bogotá*N* = 88			Cali*N* = 79			Bucaramanga*N* = 45			
	All infants	BPD *n* = 44	RR, 95% CI	All infants	BPD *n* = 14	RR, 95% CI	All infants	BPD *n* = 6	OR, 95% CI	P*
Maternal and infant baseline characteristics										
Maternal age, mean ± (SD)	27.7 (6.2)	27.8 (6.5)	1.00 (0.98; 1.02)	27.4 (6.4)	26.6 (5.6)	1.00 (0.98; 1.01)	26.4 (7.0)	23.8 (5.9)	0.99 (0.98; 1.01)	0.6607
Antenatal steroids, *n* (%)	75 (87.2)	38 (50.7)	1.15 (0.84; 1.58)	71 (89.9)	12 (16.9)	0.92 (0.70; 1.22)	38 (84.4)	6 (15.8)	1.17 (0.89; 1.53)	<0.0001
Preterm premature rupture of membranes (%)	34 (38.6)	17 (50.0)	0.99 (0.80; 1.24)	32 (40.5)	9 (28.1)	1.19 (1.01; 1.41)	10 (22.2)	4 (40.0)	1.41 (1.13; 1.75)	0.1918

Gender,*n* (%)										
Female	45 (51.1)	21 (46.7)	1	39 (49.4)	7 (17.9)	1	20 (45.5)	2 (10.0)	1	0.0018
Male	43 (48.9)	23 (53.5)	1.07 (0.87; 1.32)	40 (50.6)	7 (17.5)	1.00 (0.84; 1.18)	24 (54.5)	4 (16.7)	1.07 (0.87; 1.32)	0.0004
Gestational age at birth, wPMA, mean ± (SD)	29.0 (1.3)	29.0 (1.3)	0.99 (0.91; 1.07)	29.4 (1.4)	28.8 (1.5)	0.95 (0.89; 1.00)	29.1 (1.2)	28.2 (0.8)	0.92 (0.85; 1.00)	0.0527
Birth weight, grams, mean ± (SD)	1285.6 (290)	1231.4 (261)	1.00 (1.00; 1.00)	1273.2 (286)	1170.4 (267)	1.01 (0.97; 1.06)	1255.0 (264)	1064.2 (261)	1.00 (1.00; 1.00)	0.0380
SGA, *n* (%)	4 (4.6)	2 (50.0)	1.00 (0.60; 1.66)	5 (6.3)	1 (20.0)	1.02 (0.72; 1.45)	3 (6.7)	0 (0.00)	0.87 (0.58; 1.30)	0.5450
Apgar score 5 min, median (IR)	9 (8-9)	9 (8-9)	1.08 (0.97; 1.19)	8 (8-9)	8 (8-9)	0.94 (0.85; 1.03)	9 (8-9)	8 (7–9)	0.95 (0.87; 1.04)	0.6286
SNAP-II, mean ± (SD)	5.6 (8.4)	5.9 (8.7)	1.00 (0.99; 1.02)	5.5 (9.1)	4.7 (8.8)	1.00 (0.99; 1.01)	6.2 (9.5)	12.3 (14.0)	1.01 (1.00; 1.02)	0.8534

Respiratory management variables in the first 7 days of life										
Ventilatory support *n* (%)										
nCPAP only	70 (79.5)	30 (42.9)	1	54 (68.4)	2 (3.7)	1	38 (84.4)	5 (13.2)	1	<0.0001
nCPAP + MV	18 (20.5)	14 (77.8)	1.42 (1.10; 1.82)	25 (31.6)	12 (48.0)	1.56 (1.33; 1.82)	7 (15.6)	1 (14.3)	1.01 (0.76; 1.34)	0.0117
FIO_2_* (median values)	0.59	0.61	1.52 (1.03; 2.24)	0.46	0.43	1.28 (0.76; 2.15)	0.55	0.53	0.91 (0.55; 1.52)	0.0103
LOMV, mean ± (SD)	4.3 (1.3)	4.2 (1.4)	0.98 (0.83; 1.15)	3.3 (1.5)	2.89 (1.6)	0.94 (0.83; 1.06)	3.7 (2.52)	0 (0.00)	—	0.1729
Use of Surfactant, *n* (%)										
Very early	45 (51.1)	12 (26.7)	1	38 (48.1)	5 (13.2)	1	23 (51.1)	3 (13.0)	1	0.2115
Rescue therapy	12 (13.6)	10 (83.3)	1.76 (1.33; 2.34)	13 (16.5)	8 (61.5)	1.62 (1.32; 2.00)	2 (4.4)	0 (0.0)	0.88 (0.53; 1.46)	0.0592
No surfactant	31 (35.2)	22 (71.0)	1.56 (1.27; 1.91)	27 (34.2)	1 (3.6)	0.91 (0.77; 1.07)	20 (44.4)	3 (15.0)	1.02 (0.83; 1.26)	<0.0001

Postnatal diagnosis										
Air leak syndrome, *n* (%)	4 (4.6)	3 (75.0)	1.30 (0.78; 2.15)	6 (7.6)	3 (50.0)	1.42 (1.04; 1.93)	2 (4.4)	0 (0.0)	0.87 (0.54; 1.41)	0.2860
PDA (%)	57 (64.8)	25 (43.9)	1.16 (0.93; 1.45)	54 (68.4)	5 (9.3)	1.31 (1.10; 1.55)	37 (82.2)	4 (10.8)	1.53 (0.89; 1.49)	<0.0001
Sepsis (%)	32 (36.4)	23 (71.9)	1.38 (1.12; 1.71)	48 (60.8)	11 (22.9)	1.14 (0.96; 1.35)	29 (64.4)	5 (17.2)	1.12 (0.91; 1.37)	<0.0001

*Comparison of BPD cases among cities.

**Table 6:** Multivariate analysis exploring associations between BPD and city of birth (*n* = 208).

	OR	95% CI	
City of birth (Bogotá)^1^	1.82	1.31	2.53
City of birth (Cali)	0.80	0.46	1.39
Gender (male)	1.04	0.93	1.15
Gestational age (wPMA)	1.02	0.97	1.07
Birth weight	1.00	1.00	1.00
Antenatal steroids	1.00	0.84	1.18
Preterm premature rupture of membranes	1.18	1.05	1.33
Ventilatory support (nCPAP + MV)	1.15	1.02	1.38
Use of Surfactant (rescue)	1.36	1.10	1.68
Use of Surfactant (no surfactant)	1.19	1.06	1.33
FIO_2_ (median value)^1^	1.54	1.10	2.16
Air Leak syndrome	0.96	0.75	1.24
PDA	1.13	1.00	1.29
Sepsis	1.14	1.01	1.29

^1^OR adjusted by interaction term City × FIO_2_ (Lincom statistics). nCPAP: nasal continuous airway pressure, MV: mechanical ventilation, PDA: patent ductus arteriousus.
